# The Effect of a Rotating Medium on Bulk Acoustic Wave Polarization: From Theoretical Considerations to Perspective Angular Motion Sensor Design

**DOI:** 10.3390/s20092487

**Published:** 2020-04-28

**Authors:** Yasemin Durukan, Michail Shevelko, Aleksandr Peregudov, Ekaterina Popkova, Sergey Shevchenko

**Affiliations:** Department of Electroacoustics and Ultrasonic Technology, Saint Petersburg Electrotechnical University, 5 Prof. Popov str., 197376 Saint Petersburg, Russia

**Keywords:** bulk acoustic wave (BAW), rotation, solid-state sensitive element (SE), angular motion sensor (AMS)

## Abstract

We study the effects of medium rotation on bulk acoustic wave (BAW) propagation. For a theoretical analysis of the BAW propagation characteristics, a motion equation for the plane harmonic waves propagating orthogonal to the rotation axis of the propagation medium was analytically resolved. We found that during medium rotation, the polarization of the waves becomes elliptical with the ratio of the polarization ellipse axes explicitly proportional to the angular velocity of the medium rotation, thereby opening the way for the design of sensitive elements (SE) for perspective angular motion sensors (AMS). Next, an analytical dependence of the SE informative parameter on the Poisson’s ratio of the acoustic duct material was obtained. The rotation effect on the dispersion of BAW propagation velocity was studied. Two approaches to the perspective SE design were proposed. An experimental study of a specially designed test assembly and SE model demonstrated high correlation with theoretical predictions and provided an estimate of a potential SE. Therefore, we believe that the study of acoustic wave propagation under nonclassical conditions is a promising direction for prospective solid-state AMS on based on BAW polarization effects design.

## 1. Introduction

The propagation of acoustic waves in solid media is not only of theoretical interest, but also practical application, for example, in the development of SE for prospective solid-state AMS based on both surface and bulk acoustic wave effects [1,2,3,4,5,6,7,8,9]. The study of the physics underlying BAW propagation under motion rotation conditions dates back to the 1960s. In the first work devoted to this problem [10], the propagation features of BAW excited by a point source in an isotropic elastic medium rotating with a constant angular velocity Ω were considered. In this paper, the motion equation for a rotating medium is provided in terms of the vector and scalar potentials. However, as a result of its solution for spherical waves, no specific conclusions regarding the types and velocities of propagating waves have been formulated.

In [11], the BAW propagation features with a flat front under rotation were analyzed. The authors noted that the presence of centripetal and Coriolis accelerations in the motion equation leads to anisotropic and dispersive medium characteristics. The authors hypothesized that the phase velocity of BAW propagating in an isotropic medium depends on the ratio of the angular velocity of medium rotation Ω and the wave radial frequency ω, which is characterized by the coefficient *W*. It is also noted that in general, the eigenvectors (wave polarization) have a complex character, which indicates an elliptical polarization of the waves due to the presence of an imaginary unit in the polarization vectors ratio. For the anisotropic propagation medium, the eigenvectors have complex characteristics and it is impossible to distinguish longitudinal and transverse components in the wave displacements. In the paper of the same group [12], a special task is considered when the direction of BAW propagation is orthogonal to the rotation axis of the medium. In this case, the types of propagating waves were found to be pure transverse quasi-longitudinal and quasi-transverse waves, correspondingly. However, despite the general validity of the theoretical prediction of the complex nature of particle oscillations in propagating waves, the authors of [11,12] also did not provide any specific expressions for the velocities and polarization of the waves.

A similar case of BAW propagation along the direction orthogonal to the rotation axis was considered in [13]. The author also suggested that the polarization of the radiated wave changes under rotation, and associated it with a change in the wave-front propagation direction, and thus, also the wave vector. However, this is a mistaken assumption because the change in the spatial orientation of particle oscillations in the wave is not related to the direction of its propagation.

In [14], an analysis was performed of both BAW and SAW propagating in an elastic medium rotating at a constant angular velocity. The authors concluded that under such conditions, BAW of pure linear polarization cannot propagate, and the phase velocity of SAW depends on the angular velocity of medium rotation.

Thus, in several considered works of theoretical nature, various authors have suggested that under rotation, BAW propagating in an elastic medium lose their polarization linearity; this statement is partly true. It is worth noting that despite the fundamental nature of the aforementioned works, the authors either did not obtain specific analytical expressions describing changes in the characteristics of BAW propagating in a rotating medium or the presented relations were not completely correct.

The study of the characteristics of BAW propagating in a rotating elastic medium has also been carried out in the field of seismology. In several works [15,16,17], the effect of the Earth’s rotation on the propagation of elastic seismic waves was estimated.

The paper [15] underlines the problems from the previously considered works: the propagation of elastic waves in an isotropic solid medium, which is under rotation, was studied. The author notes that the propagation pattern directly depends on the Kibel (Rosby) number, the reciprocal of *W*, which characterizes geophysical phenomena, taking into account the Coriolis force. The types of waves existing in such an environment are determined to be quasi-longitudinal and quasi-transverse dispersive ones. At that, for small Kibel numbers, these waves are closest to pure longitudinal and pure transverse ones.

The effect of the Earth’s rotation on the propagation of seismic waves was analyzed in [16,17]. The authors note that a longitudinal wave propagating along the axis of rotation does not change its polarization, and a transverse wave is a combination of two circularly polarized waves with different velocities. This assumption was confirmed by a series of previous studies including a recent contribution by our research group [18,19].

These works aimed at investigating the possibility of applying the effects under rotation to create a solid-state SE of AMS. The theoretical results obtained in this way made it possible not only to put forward several concepts for the construction of SE, but also to confirm experimentally the possibility of developing a solid-state AMS on BAW. The authors determined the types of waves propagating under rotation for two special cases: when the direction of wave propagation and the rotation axis of the medium coincide and are orthogonal. The combination of these tasks describes the general case when these directions are arbitrarily oriented.

This approach is justified by the fact that the motion equation solution for arbitrary mutual directions of wave propagation and the rotation axis does not give a result that is convenient for analyzing and determining the characteristics of the propagating waves. Also, the solution of the task for the BAW propagation direction orthogonal to the rotation axis makes it possible to evaluate the stability of the informative parameter to the rotation along other axes.

For the first special case, several concepts were proposed, test models of the AMS SE were made and the experimental results confirmed the theoretical principles [18,19].

In this paper, the second special case is to be considered, i.e., the types of propagating waves are determined, an informative parameter is found and the concept of the AMS SE design is proposed.

## 2. Theoretical Analysis

### 2.1. Theoretical Basis

The theory of AMSSE is based on motion Equation (1) [10]:

(1) $$\rho (\frac{\partial^{2}\xi_{i}}{\partial t^{2}}+2\left(\in_{ink}\Omega_{n}\right)\frac{\partial \xi_{k}}{\partial t}+\left(\Omega_{i}\Omega_{k}\xi_{k}-\Omega_{k}\Omega_{k}\xi_{i}\right))=\frac{\partial \sigma_{ik}}{\partial x_{k}}$$

where Ω*_n_* is an angular velocity component, *ρ* is the medium density, ξ*_k_* is the displacement components, ∈*_ink_* is the Levi-Civita symbol, *σ_ik_* = *C_iklm_* *u_lm_* is the mechanical stress, elastic moduli, deformation tensors, respectively, and *x_k_* and *t* are the spacial and time coordinates. Equation (1) is given in tensor form.

The solution of motion in Equation (1) can be found for various types of waves and their propagation media. In the present paper, Equation (1) is solved for plane harmonic waves:

(2) $$\xi_{i}=\xi_{0i}\cdot \exp\left[j\left(\omega t-k_{m}x_{m}\right)\right]$$

where ξ_0*i*_ is an amplitude of particle displacement, *j* is the imaginary unit, *ω* is the radial frequency and *k_m_* is the wave vector component.

The medium in which acoustic waves are propagating can be isotropic or crystalline of any symmetry. In the present paper, the material class is limited by an isotropic medium. The Equation (1) solution is also valid for the BAW propagation along the crystallographic axes, for example, the *X*_1_ axis of a cubic symmetry crystal.

In this case, Equation (1) transforms into the Equation (3):

(3) $$\{\begin{array}{c} \left[C_{11}-\rho V^{2}\left(1+W_{2}^{2}+W_{3}^{2}\right)\right]\xi_{01}-\left(2jW_{3}-W_{1}W_{2}\right)\rho V^{2}\xi_{02}+\left(2jW_{2}+W_{1}W_{3}\right)\rho V^{2}\xi_{03}=0\\ \left[C_{44}-\rho V^{2}\left(1+W_{1}^{2}+W_{3}^{2}\right)\right]\xi_{02}+\left(2jW_{3}-W_{1}W_{2}\right)\rho V^{2}\xi_{01}-(2jW_{1}-W_{2}W_{3})\rho V^{2}\xi_{03}=0\\ \left[C_{44}-\rho V^{2}\left(1+W_{1}^{2}+W_{2}^{2}\right)\right]\xi_{03}-\left(2jW_{2}-W_{1}W_{3}\right)\rho V^{2}\xi_{01}+\left(2jW_{1}+W_{2}W_{3}\right)\rho V^{2}\xi_{02}=0 \end{array}$$

where $W_{i}=\Omega_{i}/\omega$ is relative angular velocity, and the elastic moduli tensor components are given in matrix form.

Equation (3) allows us to determine the BAW characteristics (propagation velocity and polarization type) under rotation for wave propagation along the crystallographic axes of cubic crystals and isotropic media (*C*_11_ = *λ* + 2*μ*; *C*_44_ = *μ*, where *λ* and *μ* are the Lame coefficients).

The theoretical basis of BAW propagation along *X*_1_ axis for the case when rotation axis is orthogonal to *x*_2_ or *x*_3_ axis (Ω ≡ Ω_2_ ≠ 0, Ω_1_ = Ω_3_ = 0 or *W* ≡ *W*_2_ ≠ 0, *W*_1_ = *W*_3_ = 0) was considered by the authors earlier in [19], where the BAW propagation velocities were found:

(4) $$V_{1,2}=\sqrt{\frac{\left(C_{11}+C_{44}\right)\left(W^{2}+1\right)\pm \sqrt{{\left(C_{11}+C_{44}\right)}^{2}{\left(W^{2}+1\right)}^{2}-4{\left(W^{2}-1\right)}^{2}C_{11}C_{44}}}{2\rho {\left(W^{2}-1\right)}^{2}}}$$

where *V*_1_, *V*_2_ are the propagation velocities of two waves satisfying (2).

The ratio between polarization vector components can be determined by substituting *V*_1_, *V*_2_ into Equation (3):

(5) $${\left(\frac{p_{1}}{p_{3}}\right)}_{1,2}=-j\frac{2\rho WV_{1,2}{}^{2}}{C_{11}-\rho V_{1,2}{}^{2}\left(1+W^{2}\right)}$$

**Figure 1:**
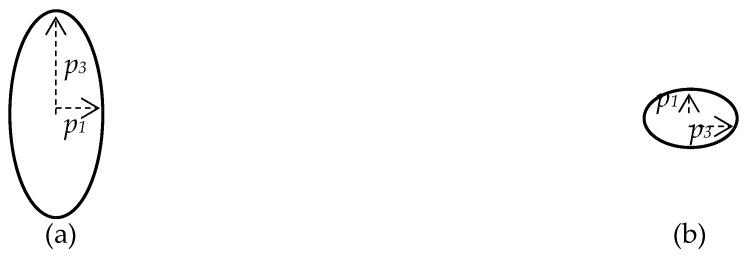
(**a**) Quasi-transverse wave; (**b**) Quasi-longitudinal wave.

As can be seen from Equations (4) and (5), the propagation velocities and the nature of the motion of particles in the wave depend on the relative angular velocity of the acoustic duct rotation. The imaginary unit in (5) corresponds to the phase shift of *π*/2 between the components of the polarization vector along the corresponding axes. Thus, particle oscillations in such waves will be elliptical. The value determined by Equation (5) corresponds to the ratio of the axes of the ellipse (Figure 1). Since, in this case, the trajectory of the particles motion of the medium does not coincide either with the propagation axis or with the orthogonal axis, such waves are of quasi-transverse (*V*_2_ = *V_qt_*) (Figure 1a) and quasi-longitudinal (*V*_1_ = *V_ql_*) (Figure 1b) types.

Equations (4) and (5) do not allow us to find analytically the functional dependences of the wave parameters on *W*. Therefore, further analysis is carried out by numerical methods for a particular acoustic duct material (any isotropic material and crystal of cubic symmetry can be used). In this paper, fused quartz was chosen for numerical analysis as it is a widely-used isotropic material for ultrasonic devices.

The dependence of the relative change in the propagation velocities of the quasi-longitudinal and quasi-transverse waves on the relative angular velocity is quadratic. Thus, the use of a change in BAW propagation velocity as an informative parameter makes SE insensitive to rotation.

### 2.2. SE Informative Parameter

**Figure 2:**
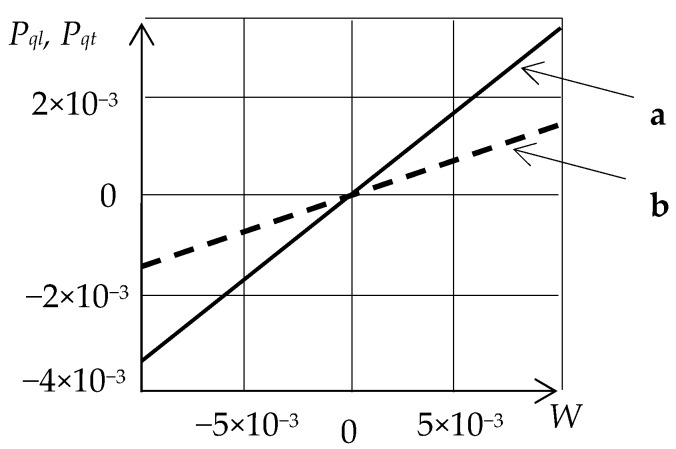
The dependence of the ratio of the ellipse polarization axes for quasi-longitudinal (**a**) and quasi-transverse (**b**) waves on the relative angular velocity.

The dependence of the ratio of the ellipse polarization axes for quasi-longitudinal $P_{ql}={\left(p_{3}/p_{1}\right)}_{ql}$ and quasi-transverse $P_{qt}={\left(p_{1}/p_{3}\right)}_{qt}$ waves on the relative angular velocity in fused quartz is shown in Figure 2.

As shown, this dependence is linear and, therefore, $P_{ql}$ and $P_{qt}$, in reference to *W*, are constant. Thus, the ratio of the ellipse polarization axes of the propagating waves can be an informative parameter for AMS SE design, with the sensitivity axis being orthogonal to the wave propagation direction.

Table 1 presents the calculation results for $P_{ql}/W$ and $P_{qt}/W$ for various materials. As shown, for a given relative velocity, the ratio of the ellipse axes lengths is inversely proportional to Poisson’s ratio *υ*.

**Figure 3:**
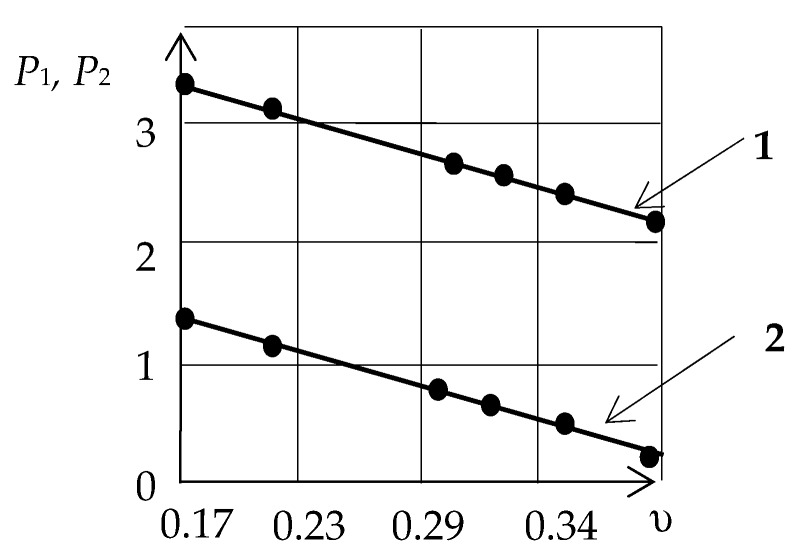
The dependence of the ratio of the ellipse’s axes on the Poisson’s ratio.

In Figure 3, the dependences of the ratio of the ellipse axes length on the Poisson’s ratio for $P_{ql}/W\;$(**1**) and $P_{qt}/W$ (**2**) are presented graphically.

The calculated dependencies can be approximated by the following functions:

(6) $$\frac{P_{ql}}{W}=\nu n_{1}+b_{1}$$

(7) $$\frac{P_{qt}}{W}=\nu n_{2}+b_{2}$$

The found values allow us to determine the nature of the particles’ motion in the medium, depending on the angular velocity for any material, according to the known Poisson’s ratio.

Equation (6) can be obtained analytically based on the known relations in terms of elastic constants λ and μ:

(8) $$C_{11}=\frac{\lambda \left(1-\mu \right)}{\mu }$$

(9) $$C_{44}=\frac{\lambda \left(1-2\mu \right)}{2\mu }$$

The numerator and denominator of Equation (5) can be divided into the product:

(10) $${\left(\frac{p_{1}}{p_{3}}\right)}_{1,2}=-j\frac{2W}{\frac{C_{11}}{\rho V_{1,2}{}^{2}}-\left(1+W^{2}\right)}$$

It can be seen that the elastic properties of the medium are taken into account only in the first term of the denominator in Equation (10). Therefore, it is expedient to consider this term separately for the phase velocity *V*_1_, at first taking into account Equation (4):

(11) $$\frac{C_{11}}{\rho V_{1}^{2}}=\frac{1}{\rho }\frac{C_{11}}{\left(C_{11}+C_{44}\right)}\frac{2\left(W^{2}-1\right)}{\left(W^{2}+1\right)+\sqrt{{\left(C_{11}+C_{44}\right)}^{2}{\left(W^{2}+1\right)}^{2}-4{\left(W^{2}-1\right)}^{2}C_{11}C_{44}}}\;=\frac{1}{\rho }\frac{2\left(W^{2}-1\right)}{\left(1+\frac{C_{44}}{C_{11}}\right)\left(W^{2}+1\right)+\sqrt{{\left(1+\frac{C_{44}}{C_{11}}\right)}^{2}{\left(W^{2}+1\right)}^{2}-4{\left(W^{2}-1\right)}^{2}\frac{C_{44}}{C_{11}}}}.$$

Thus, the influence of the elastic properties of the medium is determined by the ratio of the elastic moduli $C_{44}/C_{11}$, which can be transformed by taking into account Equation (8):

(12) $$\frac{C_{44}}{C_{11}}=\frac{\left(1-2\mu \right)}{2\mu }\frac{\mu }{\left(1-\mu \right)}=\frac{1-2\mu }{2\left(1-\mu \right)}=G$$

Then, the ratio of the ellipse polarization axes takes the form:

(13) $${\left(\frac{p_{1}}{p_{3}}\right)}_{1,2}=-\frac{j2W}{\left(\frac{1}{\rho }\frac{2\left(W^{2}-1\right)}{\left(1+G\right)\left(W^{2}+1\right)+\sqrt{{\left(1+G\right)}^{2}{\left(W^{2}+1\right)}^{2}-4{\left(W^{2}-1\right)}^{2}G}}-\left(1+W^{2}\right)\right)}$$

### 2.3. BAW Dispertion under Rotation

The assumption that the medium of wave propagation becomes dispersive under rotation is incorrect for any mutual orientation of BAW propagation direction and the rotation axis. There is no dispersion if the rotation axis coincides with the wave propagation direction, because the transverse wave propagation velocity, which is a superposition of two circularly polarized waves, does not depend on the ultrasonic wave frequency [19].

For the case when the wave propagation direction is orthogonal to the rotation axis, the relative change in the propagation velocity is proportional to *W*. The dependence of the magnitude of the relative change of the BAW propagation velocity Δ*V*/*V* for quasi-longitudinal and quasi-transverse waves on the rotation frequency Ω and BAW frequency *f* is represented in Table 2.

As shown in Table 2, such a small relative change of BAW propagation velocity will not affect the operation of the AMS SE, where the informative parameter is the magnitude of the orthogonal component of the polarization vector, which appears in the radiated wave of linear polarization under rotation.

## 3. Experimental Research

The total wave displacement propagating through the medium is a superposition of displacements in the quasi-longitudinal and quasi-transverse waves of elliptical polarization, which have different phase velocities. As a result of the interference of these waves, at certain distances from the emitter, the ratio between particle oscillations in the radiated wave and in the orthogonal direction reaches its maximum. Using the expressions for the wave propagation velocities (Equation (4)) and the ratio between the displacement vectors of these waves (Equation (5)), the level of the informative signal can be estimated. Thus, an informative parameter is the orthogonal component of the displacement vector, which appears under rotation. It can be shown that it also appears during the propagation of the excited pure transverse wave.

For SE built on this principle, it is necessary to calculate the distance at which the maximum of the orthogonal component of the polarization vector of the propagating wave is observed. It is determined from the 2*π* multiplicity condition for the phase difference of two base waves depending on time period *T*:

(14) $$k_{1}x-k_{2}x+\phi =2\pi n$$

(15) $$x_{n}=\frac{\left(n-0.5\right)TV_{1}V_{2}}{V_{2}-V_{1}}$$

**Figure 4:**
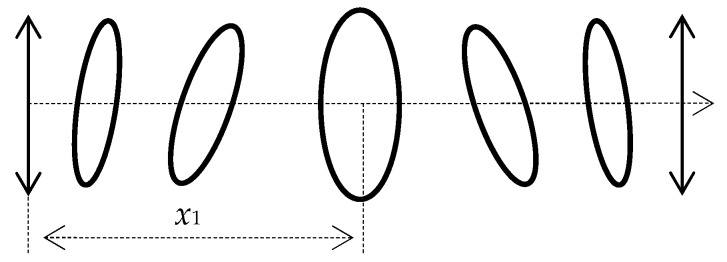
Continuous emission mode: the trajectory of particle motion in the summarized linear polarized transverse wave.

For example, for fused quartz, the distance of the first maximum at a frequency *f* = 0.5 MHz is *x*_1_ = 10.2 mm; the next maximum is observed at a distance of *x*_2_ = 30.6 mm, etc. Thus, for the continuous emission mode, if the receiver of the longitudinal component of the displacement vector is placed at a certain distance from the emitter of a pure transverse wave, then the magnitude of the longitudinal component is a criterion for determining the rotation velocity. The trajectory of particle motion in the summarized linear polarized transverse wave is shown in Figure 4.

**Figure 5:**
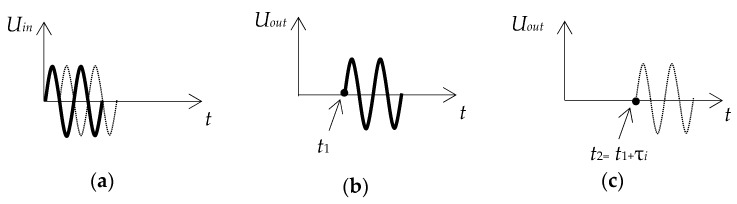
Pulse emission mode: (**a**) radiated pulse; (**b**) received pulse of quasi-longitudinal wave; (**c**) received pulse of quasi-transverse wave.

When using the pulse emission mode (Figure 5a), at a certain distance from the emitter (E), the basic waves of elliptical polarization will scatter, stop interacting and begin to propagate separately. This distance can be determined with a known pulse duration *τ_i_*. At the take-off distance at moment *t*_1_, the pulse from the quasi-longitudinal wave first appears (Figure 5b), because its propagation velocity is greater than the quasi-transverse one. At moment *t*_2_ at the same distance, i.e., *x_t_*, there will be a pulse from a slower quasi-transverse wave (Figure 5c). The waves stop interacting when the beginning of the slow-wave pulse arrives at the observation point at least *τ_i_* after the end of the fast-wave pulse. Thus, if the receiver (R) of the longitudinal wave is placed at a distance greater than the specified one, two identical signals for two longitudinal components of the displacement vectors will be received: the first one from the quasi-longitudinal wave and the second one from the quasi-transverse wave.

The condition for the separate propagation of the base waves is the delay of the quasi-longitudinal wave pulse on the pulse duration time *τ_i_*:

(16) $$\frac{x_{t}}{V_{1}}=\tau_{i}+\frac{x_{t}}{V_{2}}$$

(17) $$x_{t}=\tau_{i}\frac{V_{1}V_{2}}{V_{2}-V_{1}}$$

For the previously considered example for the acoustic duct material of fused quartz, with a pulse duration of 1 μs, the distance between E and R is to be of at least 10 mm to provide the separate signal reception.

## 4. Experimental Results

**Figure 6:**
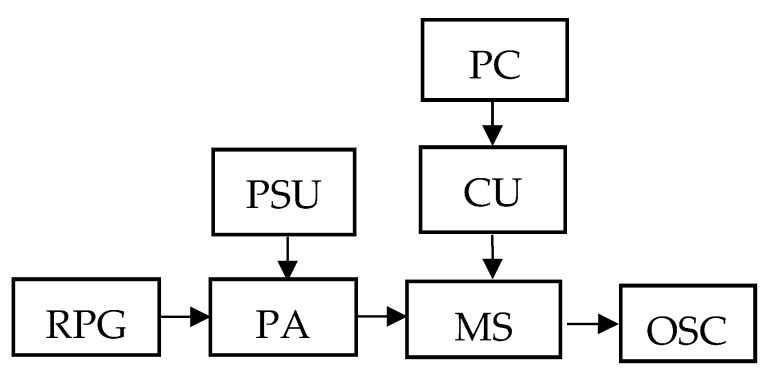
The block diagram of the experimental setup.

**Figure 7:**
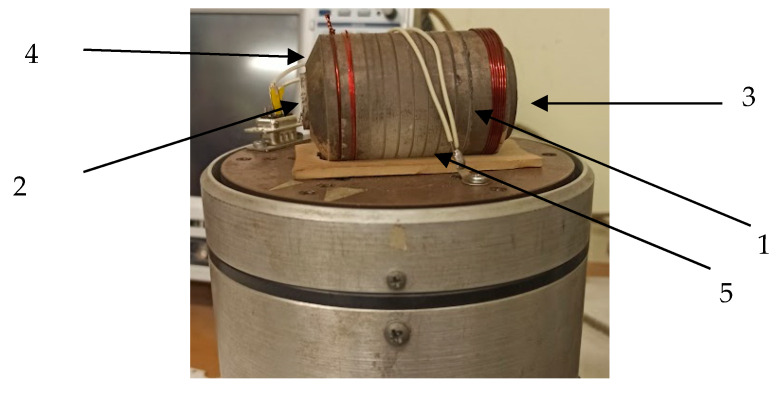
The SE test model.

The block diagram of the experimental setup is shown in Figure 6. The RF pulse generator (RPG) excites harmonic oscillations with an amplitude of 4 V at a frequency of 0.5 kHz, which are then fed to a power amplifier (PA) with a power supply unit (PSU). Oscillations amplified up to an amplitude of 200 V are fed to a measuring stand (MS), which is a uniaxial automated centrifuge [20] with the AMS SE prototype placed on it (Figure 6). The MS is controlled by a PC through the control unit (CU) with the specific software. The informative signal take-off is made using the MS built-in connector and the developed signal pickup system from the SE layout (Figure 7). The received MS signal is fed to the oscilloscope (OSC).

The SE test model is a solid-state acoustic duct 1 (Figure 7) made of fused quartz in a cylindrical shape. On its opposite plane-parallel ends, plate piezoceramic transducers 2 and 3 are placed with different polarizations. The radiating transducer 2 has a transverse polarization (T), and the receiving transducer has a longitudinal polarization (L). The acoustic duct includes facet 4, applied around the end, where the radiating transducer is placed. Facet 4 and notch 5, applied along the generatrix, lower the level of reflected (noise) signals by 20 dB and reduce the decay time. This makes it possible to operate at a higher pulse transmission frequency and, thus, to extend the number of process implementations to average an informative signal, which increases the measurement accuracy.

The output voltage of the AMS SE layout is determined by the following relation:

(18) $$U_{\mathrm{out}}=U_{\mathrm{in}}K_{\mathrm{acoustic}}K_{gyro}\Omega$$

where $U_{\mathrm{in}}$ is the voltage at the SE input, $K_{\mathrm{acoustic}}$ is the transmission coefficient of the acoustic path of the SE and $K_{gyro}$ is the transmission coefficient of the gyroscopic component $P_{ql}/W$.

For the developed layout, $U_{in}=200\;V,\;K_{\mathrm{acoustic}}=-8\;\mathrm{dB},\;\mathrm{and}\;K_{gyro}=\left(3.3/2\pi \right)W=4.23\times 10^{-6}$. The parameters of the emitting (E) and receiving (R) transducers are presented in Table 3.

**Figure 8:**
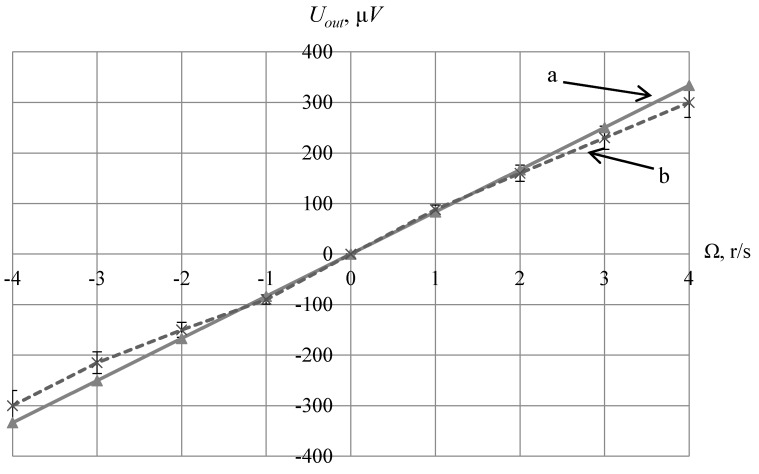
The dependence of the output voltage on the angular velocity of rotation:theoretical (**a**) and experimental (**b**) results.

The acoustic duct was made of 70-mm-long fused quartz, and salol was used as the contact layer material between the acoustic duct and transducers. In the calculations, the load of the receiving piezoelectric plate on the shunt resistance of 50 Ohms was taken into account. The results of the dependence of the output voltage *U_out_* on the angular velocity of rotation Ω, calculated theoretically and obtained during the experiment, are presented in Figure 8.

## 5. Discussion

As shown in Figure 8, the obtained experimental values of the output voltage have good correlation with the calculated values within the measurement error. It is worth noting that this SE model has large dimensions, which precludes its use in products with critical size requirements. The dimension reduction of the SE layout for this construction concept cannot be performed since it will increase the operating frequency, which will proportionally reduce the level of the informative signal. This is due to the inversely proportional dependence of the coefficient of the gyroscopic component on the ultrasonic frequency *f*. In this regard, the use of this method to measure the angular velocity is possible not as an AMS SE, but directly, when placed on structural parts of an object. For devices that correspond to miniaturization trends, the most promising is the SE built on the principle of detecting the polarization vector rotation in the transverse wave radiated into the direction that coincides with the rotation axis. Thus, to detect rotation along the axis oriented arbitrarily to the direction of BAW propagation, three SE or one SE for which it is possible to place three pairs of transducers on mutually-orthogonal planes can be used.

## 6. Conclusions

Regarding the features of BAW propagation in the direction orthogonal to the axis of medium rotation, the following conclusions can be made:

1. It is advisable to use the magnitude of the orthogonal component of the polarization vector in the radiated linear polarized wave as an informative parameter.
2. The value of the informative parameter is dependent on the Poisson’s ratio *υ* of the BAW propagation medium; the larger the *υ* value, the lower the level of the informative signal.
3. The dispersion of the phase velocity of BAW propagation is not observed when the wave propagates in the direction coinciding with the rotation axis, or when the BAW propagates orthogonal to the rotation axis with a displacement vector coinciding with the rotation axis. In other cases, there is a dispersion of the BAW phase velocity.
4. The AMS SE based on the principles of detecting the rotation of the radiated transverse wave polarization vector is uniaxial. The influence of rotation around the axis orthogonal to the BAW propagation direction is a second-order small quantity, and thus, can be neglected when determining the angular velocity.
5. The theoretical assumptions, as well as the proposed concept for SE design, are in agreement with the experimental results demonstrating the linear-type dependence of the informative parameter on the angular velocity of medium rotation.
6. The level of the informative signal, obtained experimentally, shows a high correlation with the previously determined theoretical sensitivity level of the proposed SE model.

## 7. Patents

Durukan, Y.; Peregudov, A.N.; Shevelko, M.M. Ultrasonic method for measuring the angular velocity. **18.02.2020,** Patent of Russian Federation *No 2714530*.

## Figures and Tables

**Table 1:** The calculation results.

Material	*υ*	*ρ* (kg/m^3^)	$P_{ql}/W$	$P_{qt}/W$
Fused quartz	0.17	2.2 × 10^3^	3.322	1.322
F1 glass	0.22	3.57 × 10^3^	3.139	1.139
Flint glass	0.30	4.76 × 10^3^	2.795	0.795
Bismuth	0.33	9.80 × 10^3^	2.667	0.667
Aluminum	0.36	2.70 × 10^3^	2.578	0.578
Plexiglass	0.40	1.15 × 10^3^	2.398	0.398

**Table 2:** The relative change of BAW propagation velocity Δ*V*/*V* × 10^9^.

		*f* (MHz)	
Ω (r/s)	0.1	0.5	1
0.5	0.0375	0.0015	0.000375
1	0.15	0.006	0.015
2	0.6	0.024	0.006
5	3.75	0.156	0.0375

**Table 3:** Parameters of emitting and receiving transducers.

Parameter	E Transducer	R Transducer
polarization type	transverse	longitudinal
Material	ZTS-19	ZTS-19
Shape	round	round
diameter (mm)	20	35
thickness (mm)	1.76	3.7
resonant frequency (MHz)	0.5	0.5
